# Transcriptional and phenotypic alterations of regulatory and effector T cells in type 1 diabetes mellitus

**DOI:** 10.1186/s43556-026-00636-9

**Published:** 2026-10-24

**Authors:** Yutong Li, Xinhui Zhao, Mingxin Xie, Eyad Elkord

**Affiliations:** 1 Centre of Excellence for Oncology and Immunology, Academy of Life and Natural Sciences, Xi’an Jiaotong-Liverpool University, No. 111 Ren’ai Road, Suzhou, 215123 China; 2 Biomedical Research Center, School of Science, Engineering and Environment, University of Salford, Manchester, M5 4WT UK

**Keywords:** Type 1 diabetes mellitus, Tregs, Transcriptional profiling, Immune checkpoints, Inflammation

## Abstract

Type 1 diabetes mellitus (T1DM) is characterized by the destruction of pancreatic β-cells, in which the dysfunction of regulatory T cells (Tregs) contributes to impaired immune tolerance. However, Treg heterogeneity remains incompletely understood, which limits the effectiveness of current therapies. Here, we have integrated transcriptome, flow cytometry, glycemic correlation, and cross-model validation to characterize the dysregulation of Tregs and effector T cells (Teffs) in a streptozotocin (STZ)-induced T1DM mouse model. Tregs exhibited a proliferative, effector-like, unstable lineage, and metabolic reprogramming features, marked by increased *Melk*, *Prf1*, *Gzma* and *Hk2*, and decreased expression of *Tnfsf15*, *Irak3* and *Zeb2* in T1DM, and alterations in the transcription-factor networks involving *Nfatc2*, *Elk1*, and *Ahr*. Flow cytometry further revealed that the frequency of splenic Tregs was significantly increased, accompanied by broad upregulation of programmed cell death protein 1 (PD-1), T cell immunoreceptor with Ig and ITIM domains (TIGIT), lymphocyte-activation gene 3 (LAG3), and T-cell immunoglobulin and mucin domain-containing protein 3 (TIM3). Meanwhile, expression of glycoprotein A repetitions predominant (GARP) and latency-associated peptide (LAP) was decreased, whereas interleukin (IL)- 17 and interferon-γ (IFN-γ) secretion were increased, suggesting a Treg phenotype with high checkpoint expression and inflammatory features. Cross-model analyses identified concordant IL-15-, IL-36 A-, E2F-, and metabolic programs across the C57BL/6, non-obese diabetic (NOD), and human datasets, including shared genes *Smad3*, *Mki67*, and *Cxcr4*. Teffs also displayed a hyperactivated but transcriptionally imbalanced phenotype in T1DM. Collectively, our findings reveal transcriptional and phenotypic alterations in Tregs and Teffs, providing new insights into T-cell dysregulation and potentially informing the development of Treg-based immunotherapies in T1DM.

**Supplementary Information:**

The online version contains supplementary material available at https://doi.org/10.1186/s43556-026-00636-9.

## Introduction

Type 1 diabetes mellitus (T1DM) is a chronic autoimmune disease in which pancreatic β-cells are damaged, resulting in insulin deficiency and metabolic disorders [1]. The incidence of T1DM is rapidly increasing worldwide. In 2025, the number of patients reached 9.5 million, and it is expected to increase to 14.7 million by 2040 [2, 3]. Although insulin injection therapy has certain efficacy, it is still insufficient to halt the progression of the disease or prevent long-term complications [4, 5]. In this case, developing immunotherapy is necessary to target immune dysfunction in T1DM. Recently, immunomodulatory therapies [6], such as Teplizumab [7] and Abatacept [8, 9], have shown preliminary effects in early clinical applications, but the long-term efficacy remains to be improved. Therefore, a comprehensive understanding of the basis of immune imbalance is needed to develop more effective therapeutic strategies for T1DM.

Regulatory T cells (Tregs) are a subset of CD4⁺ T cells that are crucial for suppressing autoimmune responses and maintaining self-tolerance [10]. Early studies have reported that defects in the quantity or function of Tregs are associated with various autoimmune diseases, including T1DM [11, 12]. Although preclinical studies have shown that expanding Tregs can alleviate pancreatic inflammation and preserve the number of β cells [13, 14], therapies based on Treg expansion still face challenges at the clinical stage for T1DM [15, 16]. A major obstacle is the considerable heterogeneity of Tregs, encompassing dynamic and context-dependent phenotypes and functional properties [17]. Thus, a comprehensive understanding of the immune regulatory mechanisms underlying Treg heterogeneity in T1DM is essential for effective immunotherapies.

Immune checkpoint molecules, such as programmed cell death protein 1 (PD-1), T cell immunoreceptor with Ig and ITIM domains (TIGIT), lymphocyte-activation gene 3 (LAG3), and T-cell immunoglobulin and mucin domain-containing protein 3 (TIM3), have emerged as key regulatory factors influencing T cell activation and tolerance [18, 19]. Although these molecules are usually associated with inhibitory signal transduction and T cell exhaustion, their roles in Tregs are more complex [20, 21]. In Tregs, the expression of immune checkpoints may reflect chronic activation, compensatory regulation, or dysfunction [22, 23]. Notably, the mechanisms by which these immune checkpoints interact with intracellular signaling pathways and drive Treg dysfunction in the autoimmune environment remain unclear. Beyond checkpoint-mediated signaling, migratory ability is also one of the key factors determining the function of T cells. Chemokine receptors could direct the localization of Tregs between lymphoid and inflamed tissues [24, 25]. In parallel, metabolic reprogramming provides an additional layer of control, and its disruption can impair the suppressive capacity of Tregs, thereby contributing to immune dysregulation [26–29], and further exacerbating immune imbalance.

In this study, we investigated the transcriptional and phenotypic alterations of Tregs and Teffs in the streptozotocin (STZ)-induced T1DM mouse model. Tregs exhibited significant transcriptional remodeling, proliferative and metabolic stress features, accompanied by loss of lineage stability and suppressive capacity. Mechanistically, a coordinated transcription factor (TF) network centered on nuclear factor of activated T cells 2 (*Nfatc2)*, ETS transcription factor (*Elk1)* and aryl hydrocarbon receptor (*Ahr*) was involved in inflammation, cell cycle, and metabolic disorders. Despite numerical expansion, Tregs displayed a suppressive-function-associated phenotype and inflammatory reprogramming, evidenced by reduced glycoprotein A repetitions predominant (GARP)/latency-associated peptide (LAP), and elevated interleukin (IL)− 17 and interferon-γ (IFN-γ) in T1DM. In parallel, Teffs remained in a hyperactivated phenotype with sustained inflammatory and cytotoxic capabilities. Overall, this study provides insights into the mechanisms of Treg dysregulation and proposes the immune checkpoint-inflammation-metabolism axis as a potential target for restoring immune tolerance in T1DM.

## Results

### Transcriptional profiling identifies distinct Treg and Teff signatures in T1DM

**Fig. 1:**
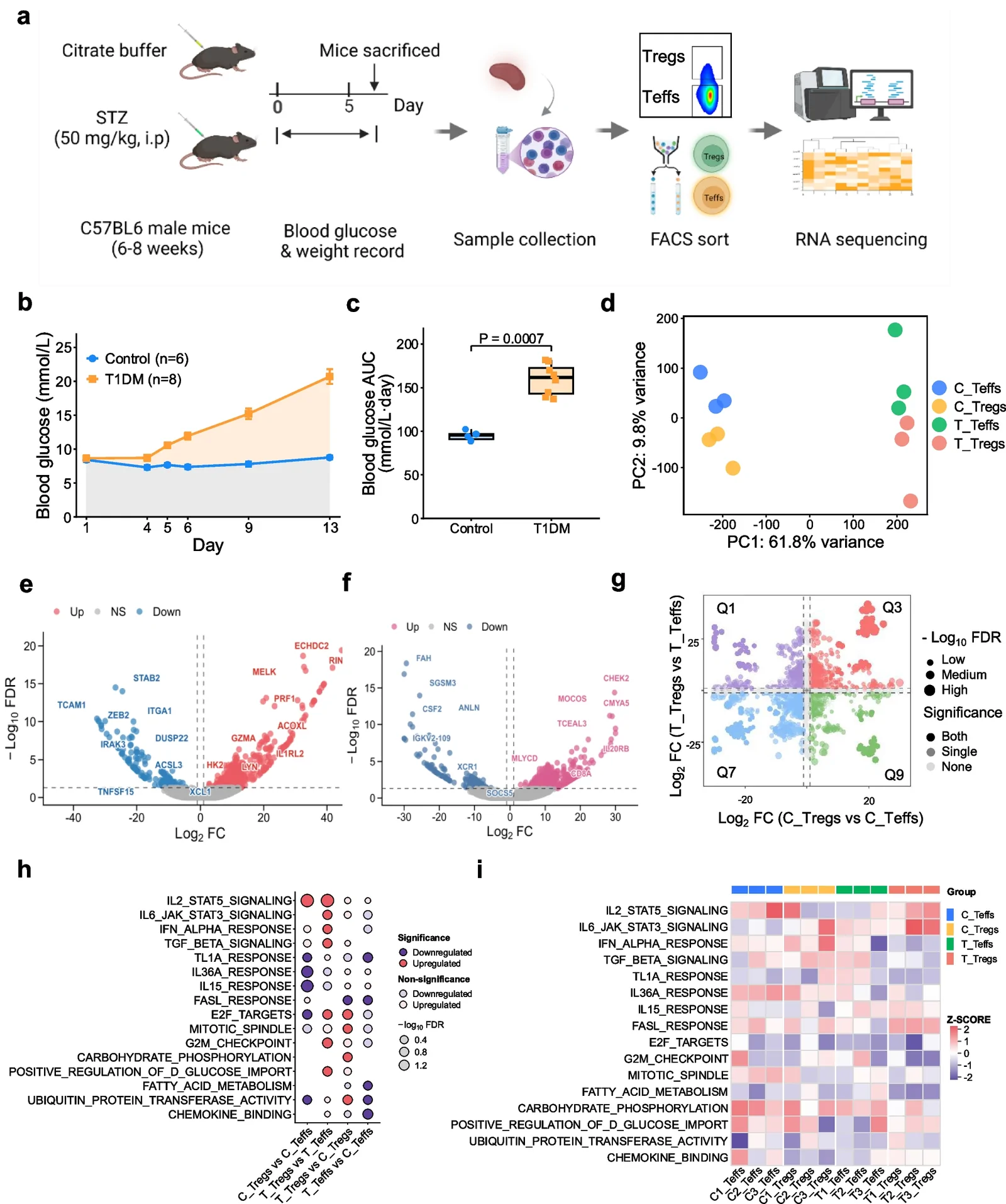
Experimental design and transcriptomic profiling of Tregs and Teffs in STZ-induced T1DM C57BL/6 mice. **a** Schematic overview of the establishment of the STZ-induced T1DM C57BL/6N mouse model, followed by splenocyte isolation, FACS sorting of Tregs and Teffs and bulk RNA-seq. **b** Line graphs showing the longitudinal change in fasting blood glucose. The points and error bars represent the mean ± SEM. The shaded area indicates the region under the mean glucose curves for each group; the gray area represents the overlapping part between the two groups. **c** Blood-glucose AUC was calculated using the trapezoidal rule and the actual intervals between measurements. Each point represents one mouse. Box plots show the median (center line), interquartile range (box), and whiskers extending to values within 1.5 × the interquartile range. **d** PCA plot based on all expressed genes of Tregs and Teffs between control and T1DM groups. **e–f** Volcano plots displaying DEGs comparing Tregs and Teffs in T1DM with control group. Significantly upregulated genes are shown in red and downregulated genes in blue (|log_2_FC| > 1 and FDR < 0.05). **g** Nine-quadrant volcano plot illustrating DEGs between Tregs and Teffs in control and T1DM conditions. The x-axis and y-axis represent the log_2_FC of Tregs vs Teffs under control and T1DM conditions, respectively. Quadrants define expression patterns: Q1 (Control down, T1DM up), Q2 (Control no change, T1DM up), Q3 (Control up, T1DM up), Q4 (Control down, T1DM no change), Q5 (no change), Q6 (Control up, T1DM no change), Q7 (Control down, T1DM down), Q8 (Control no change, T1DM down), Q9 (Control up, T1DM down). **h** Bubble plot summarizing significantly enriched gene sets from GSEA, including mouse-ortholog hallmark gene sets, GO terms and immunologic signature gene sets across comparisons. Bubble size corresponds to -log_10_ FDR, and color indicates NES. **i** GSVA enrichment heatmap of GSEA-identified gene sets across individual samples. Colors represent z-scored GSVA enrichment values. Created with BioRender.com

We established a multiple low-dose STZ-induced T1DM model in male C57BL/6 N mice aged 6–8 weeks, isolated splenic Tregs and Teffs by fluorescence-activated cell sorting (FACS) and subjected the sorted cells to bulk RNA sequencing (RNA-seq) (Fig. 1a). STZ-treated mice developed progressively increasing blood glucose levels (Fig. 1b). Terminal blood glucose was 22 ± 3 mmol/L in the STZ group and 9 ± 1 mmol/L in the control group (Fig. 1b). STZ-treated mice also exhibited a significantly higher blood glucose area under the curve (AUC) than control mice (Fig. 1c). In addition, these mice showed progressive body-weight loss (Fig. S1a), a reduced body-weight AUC (Fig. S1b), and a negative correlation between cumulative blood glucose and body weight (Fig. S1c). Principal component analysis (PCA) revealed distinct clustering patterns of Tregs and Teffs between control and T1DM groups, with PC1 accounting for 61.8% of the variance (Fig. 1d). Sample-to-sample correlation analysis further supported the separation among the experimental groups (Fig. S2a).

Differentially expressed gene (DEG) analysis revealed 812 significantly upregulated and 258 downregulated genes in splenic Tregs in T1DM compared with controls (Fig. 1e). Upregulated genes were associated with proliferation (*Melk*, *Rin1*), cytotoxicity (*Prf1*, *Gzma*), inflammatory signaling (*Il1rl2*), and metabolic remodeling (*Hk2*, *Acoxl*, *Echdc2*). Conversely, genes involved in immunosuppression (*Tnfsf15*, *Irak3*, *Dusp22*), lineage stability (*Zeb2*), tissue homing (*Itga1*, *Plxna1*), metabolic fitness (*Acsl3*, *Bdh2*), and stress adaptation (*Hspa1a, Ifi44*) were significantly downregulated*.* In parallel, splenic Teffs in T1DM displayed 744 significantly upregulated and 309 downregulated genes compared with controls (Fig. 1f). Upregulated genes included those associated with metabolic adaptation (*Mocos*, *Mlycd*), DNA damage responses (*Chek2*), effector identity (*Cd8a*), inflammatory signaling (*Il20rb*), and transcriptional regulation (*Tceal3*). Conversely, downregulation of *Fah*, *Sgsm3*, *Anln*, and *Xcr1* indicated the alterations in metabolism, signal transduction, cell cycle progression, and migratory capacity of Teffs in T1DM.

To further examine cell-type-specific transcriptional changes, we performed a two-dimensional quadrant analysis (Fig. 1g). Notably, quadrant 3 (Q3) contained genes expressed at higher levels in Tregs than in Teffs under both control and T1DM conditions, such as *Ctla4*, *Il1r2*, *Gpr83*, and *Tnfrsf4*. In contrast, Q9 contained genes expressed at higher levels in Tregs than in Teffs under control conditions but at lower levels under T1DM conditions, such as *Chek2*, *Cmya5*, and *Tcam1*. Meanwhile, Q7 contained genes preferentially enriched in Teffs. These genes were associated with cytotoxicity (*Prf1*), cytokine signaling (*Il21*, *Il4*), and NK-like receptor signaling (*Klrc1*, *Klrd1*). The DEG heatmap further illustrated these expression patterns (Fig. S2b). In particular, Tregs from T1DM mice showed significant upregulation of genes associated with cellular regulation and metabolism, including *Foxo4*, *Hk2*, and *Ppargc1b*.

Collectively, Tregs in T1DM exhibited increased expression of proliferation-, effector-, and metabolism-associated genes, alongside reduced expression of genes involved in immunoregulation, lineage maintenance, tissue homing, metabolic fitness, and stress responses. Meanwhile, Teffs exhibited enhanced proliferative and inflammatory programs and dysregulated metabolic and migratory features. These alterations may act synergistically to cause immune dysregulation in STZ-induced T1DM.

### GSEA and GSVA reveal inflammatory, proliferative, and metabolic remodeling in Tregs and Teffs

**Fig. 2:**
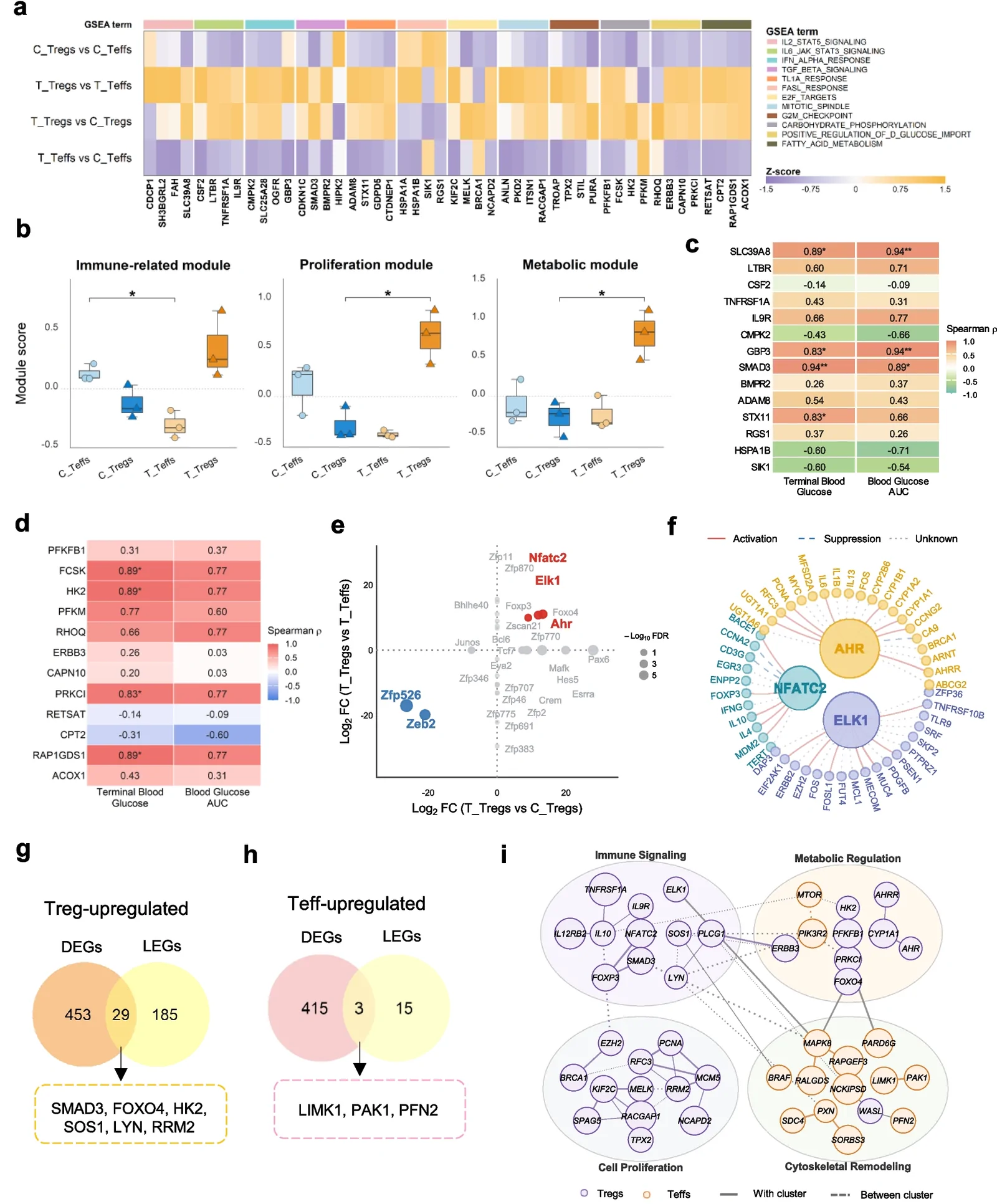
Correlation and network analysis in Tregs and Teffs. **a** Heatmap of representative LEGs from GSEA terms related to cytokine and Treg signaling, apoptosis, cell-cycle regulation, glucose metabolism and fatty-acid metabolism. **b** Box plots of LEG module scores reveal cell-type-specific transcriptional changes in T1DM. **c** & **d** Heatmap of Spearman correlations between immune-, proliferative- and metabolic-associated LEGs of Tregs and glycemic severity (terminal blood glucose and blood glucose AUC). **e** Quadrant plot of TFs among significant DEGs of Tregs. Each dot represents a TF, plotted by log_2_FC in Tregs in T1DM vs control (x‑axis) and Tregs in T1DM vs Teffs in T1DM (y‑axis). **f** Network diagram illustrating interactions between upregulated TFs and their predicted target genes in Tregs based on TRRUST database. **g** & **h** Venn diagrams illustrating overlapping genes between upregulated DEGs and LEGs from GSEA in Tregs and Teffs. **i** Protein–protein interaction (PPI) network of genes upregulated in Tregs and Teffs in T1DM, comprising 46 connected proteins and 56 interactions (combined score ≥ 0.70); node outlines indicate lineage, edge width indicates interaction confidence, edge style indicates Markov clustering algorithm (MCL) clustering, and ellipses denote visualization-only functional annotations

To characterize potential functional alterations at the transcriptional level, we further performed GSEA across four comparisons (Fig. 1h) and GSVA across individual samples (Fig. 1i). We found that the IL-2-STAT5 signaling pathway activity was significantly enriched in Tregs relative to Teffs under both control and diabetic conditions (Fig. 1h). However, its enrichment did not differ significantly in Tregs between control and T1DM conditions (Fig. S3a), indicating preservation of this core Treg-associated program rather than disease-specific activation. In contrast, pathways related to inflammatory signaling, such as IL-6-JAK-STAT3 and IFN-α-response gene sets, were preferentially enriched in Tregs under T1DM (Fig. 1h), reflecting their Teff-like characteristics and enhanced cytokine responsiveness. Tumor necrosis factor-like ligand 1 A (TL1A) [30], IL-36 A [31], and IL-15 [32] -response signatures were negatively enriched in Tregs relative to Teffs under control conditions (Fig. 1h & Fig. S3b). However, IL-36 A- and IL-15-response shifted toward enrichment in Tregs in T1DM. GSVA across individual samples also supported these enrichment patterns (Fig. 1i). The heatmap of representative leading-edge genes (LEGs) further demonstrated coordinated changes across the four comparisons (Fig. 2a). In addition, the immune-module score decreased significantly in Teffs, whereas it increased in Tregs in T1DM (Fig. 2b).

Apoptosis- and cell-cycle-associated transcriptional programs were also altered in Tregs and Teffs. The Fas ligand (FasL)-response signature showed significant negative enrichment in both Tregs and Teffs in T1DM (Fig. 1h). Notably, Tregs in T1DM also showed positive enrichment of E2F targets (Fig. S3c) and mitotic-spindle programs, together with an upward trend in the G2M-checkpoint program (Fig. 1h). LEGs related to cell cycle, including *Kif2c*, *Melk*, *Tpx2*, and *Racgap1*, showed pronounced expression changes in Tregs from T1DM mice (Fig. 2a). Consistently, the proliferation-module score was elevated significantly in Tregs in T1DM (Fig. 2b). No similar upregulation was detected in Teffs.

Metabolic reprogramming also displayed cell-type-specific alterations in T1DM. In Tregs, carbohydrate-phosphorylation and positive-regulation-of-D-glucose-import programs were significantly enriched (Fig. 1h), with representative LEGs including the glycolysis-associated genes *Hk2*, *Pfkfb1*, *Pfkm*, and *Prkci* (Fig. 2a). In contrast, the fatty acid metabolism gene set showed significant negative enrichment in Teffs (Fig. 1h). The metabolic-module score increased significantly in Tregs in T1DM, whereas no significant change was detected in Teffs (Fig. 2b).

Next, we examined associations between immune-, proliferation-, and metabolism-associated LEGs and the burden of blood glucose. For instance, the expression levels of *Slc39a8* and *Gbp3* were positively correlated with blood glucose AUC, while *Smad3* expression was positively correlated with terminal blood glucose (Fig. 2c). Among the metabolic LEGs, *Fcsk*, *Hk2*, and *Rap1gds1* showed significant positive correlations with terminal blood glucose (Fig. 2d).

### Integrated TF-protein–protein interaction (PPI) networks link regulatory modules in Tregs and Teffs in T1DM

We next characterized disease-associated changes in TF expression. In Tregs, *Nfatc2*, *Elk1*, and *Ahr* were significantly upregulated, whereas *Zfp526* and *Zeb2* were significantly downregulated in T1DM (Fig. 2e). In Teffs, four zinc‑finger TFs (*Zfp707*, *Zfp346*, *Zfp46*, and *Zfp775*) were upregulated in T1DM (Fig. S4a).

Predicted TF associations and target genes suggested potential links between *Nfatc2*, *Elk1*, and *Ahr* and activation-, proliferation-, and environmental-response programs in Tregs. *Nfatc2* was associated with inflammatory and immune regulatory TFs (*Nfkb1*, *Stat1/3/5 a*, *Irf4*, and *Jun*), as well as factors related to proliferation and stress (*Myc*, *Tp53*, and *Parp1*) (Fig. S4b). TF-target analyses identified potential *Nfatc2* targets involved in immunomodulation, including *Foxp3*, *Il10*, *Il4*, and *Ifng* (Fig. 2f). *Elk1* also linked proliferative and inflammatory programs through interactions with *E2f1*, *Creb1*, *Srf*, and *Rela* (Fig. S4b), and regulation of target genes involved in cell cycle and signal transduction, such as *Skp2*, *Erbb2*, and *Fos* (Fig. 2f). Meanwhile, *Ahr* interacted with TFs involved in inflammation (*Nfkb1*, *Stat1/3/6*), chromatin remodeling (*Ep300*, *Smarca4*), and metabolic TFs (*Ppara*, *Hnf4a*) (Fig. S4b). *Ahr* upregulation in Tregs was predicted to activate target genes, including metabolic enzymes (*Cyp1a1*, *Cyp1b1*, *Ugt1a1*) and cell cycle regulators (*Ccng2*, *Pcna*), as well as inflammatory mediators (*Il6*, *Il1b*, *Il13*) (Fig. 2f).

Subsequently, we constructed a STRING PPI network using genes shared between the upregulated DEGs-LEGs in Tregs (Fig. 2g) and Teffs (Fig. 2h), together with predicted targets of TFs in Tregs (Fig. 2i). This network was divided into four biological annotation regions, representing immune signal transduction, metabolic regulation, cell proliferation, and cytoskeletal remodeling. Within the upregulated network, PLCG1, RACGAP1, RRM2, and IL10 were prominent nodes among the Treg-associated candidates. MAPK8 and PIK3R2 were important connecting proteins related to Teffs. The PLCG1-LYN-SOS1 axis connected the immune signal transduction with metabolic regulatory factors, while KIF2C, MELK, RACGAP1, RRM2, MCM5, and RFC3 formed a sub-network related to proliferation. AHR, HK2, and PFKFB1 linked TFs to the metabolic programs. Collectively, these network associations supported prioritization of *Plcg1*, *Racgap1*, *Rrm2*, *Kif2c*, *Melk*, *Ahr*, and *Hk2* as candidates for further investigation. We also used downregulated candidate genes in Tregs (Fig. S4c) and Teffs (Fig. S4d) to conduct a separate PPI network (Fig. S4e). Eight genes were shared between Tregs and Teffs, including *Cxcl10*, *Xcl1*, *Dusp1*, *Fos*, *Jun*, *Junb*, *Hspa1a*, and *Hspa1b.*

Together, the networks derived from upregulated and downregulated candidates jointly highlighted the complementary alterations in immune signal transduction, proliferation, metabolism, stress response, and cellular organization in Tregs and Teffs under STZ-induced T1DM conditions.

### Treg expansion accompanies broad immune-checkpoint upregulation in T1DM

**Fig. 3:**
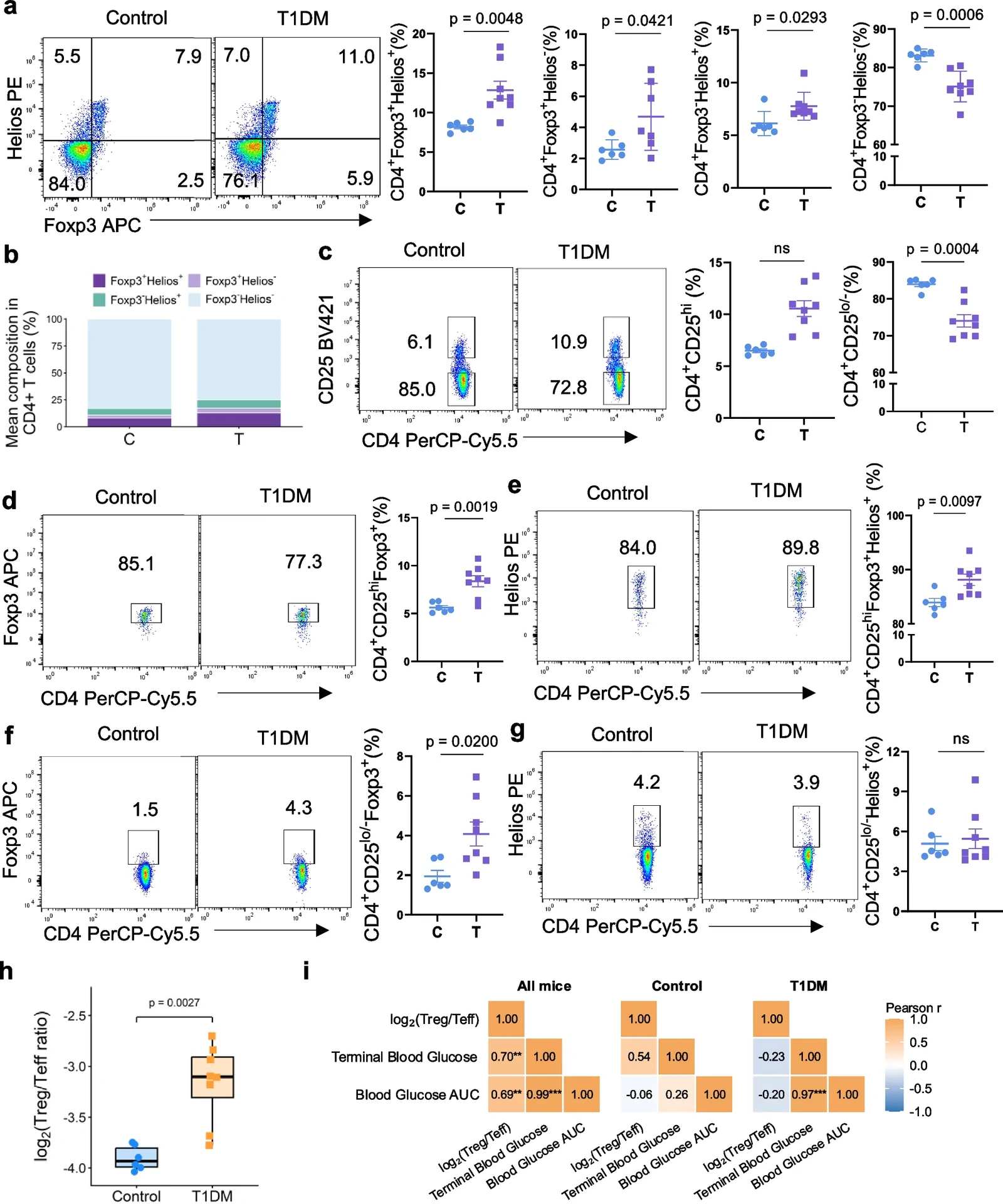
Lineage marker expression of Tregs and Teffs. **a** Representative flow cytometry plots and bar plots of the proportions of Foxp3^+^Helios^+^, Foxp3^+^Helios^−^, Foxp3^−^Helios^+^, Foxp3^−^Helios^−^ in CD4^+^ T cells in control (*n* = 6) and T1DM (*n* = 8) groups. **b** Stacked bar plots showing the mean composition of Foxp3/Helios-defined CD4⁺ T-cell subsets in control and T1DM groups. **c** Representative flow cytometry plots and bar plots of the frequency of CD3^+^CD4^+^CD25^hi^ and CD3^+^CD4^+^CD25^lo/−^ T cells. **d** Representative flow cytometry plots and bar plots of the frequency of Tregs (CD3^+^CD4^+^CD25^hi^Foxp3^+^). **e** Representative flow cytometry plots and bar plots showing the frequency of Helios in Tregs. **f** & **g** Representative flow cytometry plots and bar plots showing the frequency of Foxp3 and Helios in Teffs. **h** Box plots showing the Treg/Teff ratio, calculated as log_2_(Tregs/Teffs), in control and T1DM groups. Each point represents one mouse; boxes indicate the median and interquartile range. **i** Pearson correlation heatmaps showing associations among the log_2_(Treg/Teff) ratio, terminal blood glucose, and glucose AUC in all mice (*n* = 14), control mice (*n* = 6), and T1DM mice (*n* = 8). Error bars represent SEM; *p* values were calculated by two-tailed Student’s t-test. *p < 0.05, **p < 0.01, ***p < 0.001

To determine whether the transcriptional alterations were accompanied by changes in immune-cell composition and phenotype, we performed multiparameter flow cytometry analyses based on the gating strategy (Fig. S5) and fluorescence-minus-one (FMO) controls (Fig. S6). In T1DM, the proportion of Foxp3⁺Helios⁺ T cell populations within CD4⁺ T cells was elevated (Fig. 3a). Consistently, the mean proportion of Foxp3⁺Helios⁺ T cells increased from 8.19% in controls to 12.84% in T1DM (Fig. 3b). In parallel with the upregulation of cell cycle- and IL-2-STAT5-associated transcriptional programs, we also observed the frequency of CD4^+^CD25^hi^Foxp3^+^ Tregs was significantly increased in T1DM (Fig. 3c & d), with enhanced Helios expression (Fig. 3e). In addition, we found that the expression of Foxp3 increased, whereas the expression of Helios remained unchanged in Teffs in T1DM (Fig. 3c, f & g). These phenotypic alterations were accompanied by a significant shift in the ratio of Tregs and Teffs (Fig. 3h). The median Treg/Teff ratio increased approximately 1.78-fold in T1DM (*p* = 0.0027).

We further evaluated the correlation between the ratio of Tregs and Teffs and blood glucose burden. Across all mice, the log_2_(Treg/Teff) was positively correlated with terminal blood glucose and blood-glucose AUC (Fig. 3i). However, neither association reached statistical significance when control and T1DM mice were analyzed separately (Fig. 3i). Thus, the combined association was mainly driven by the differences between the experimental groups.

**Fig. 4:**
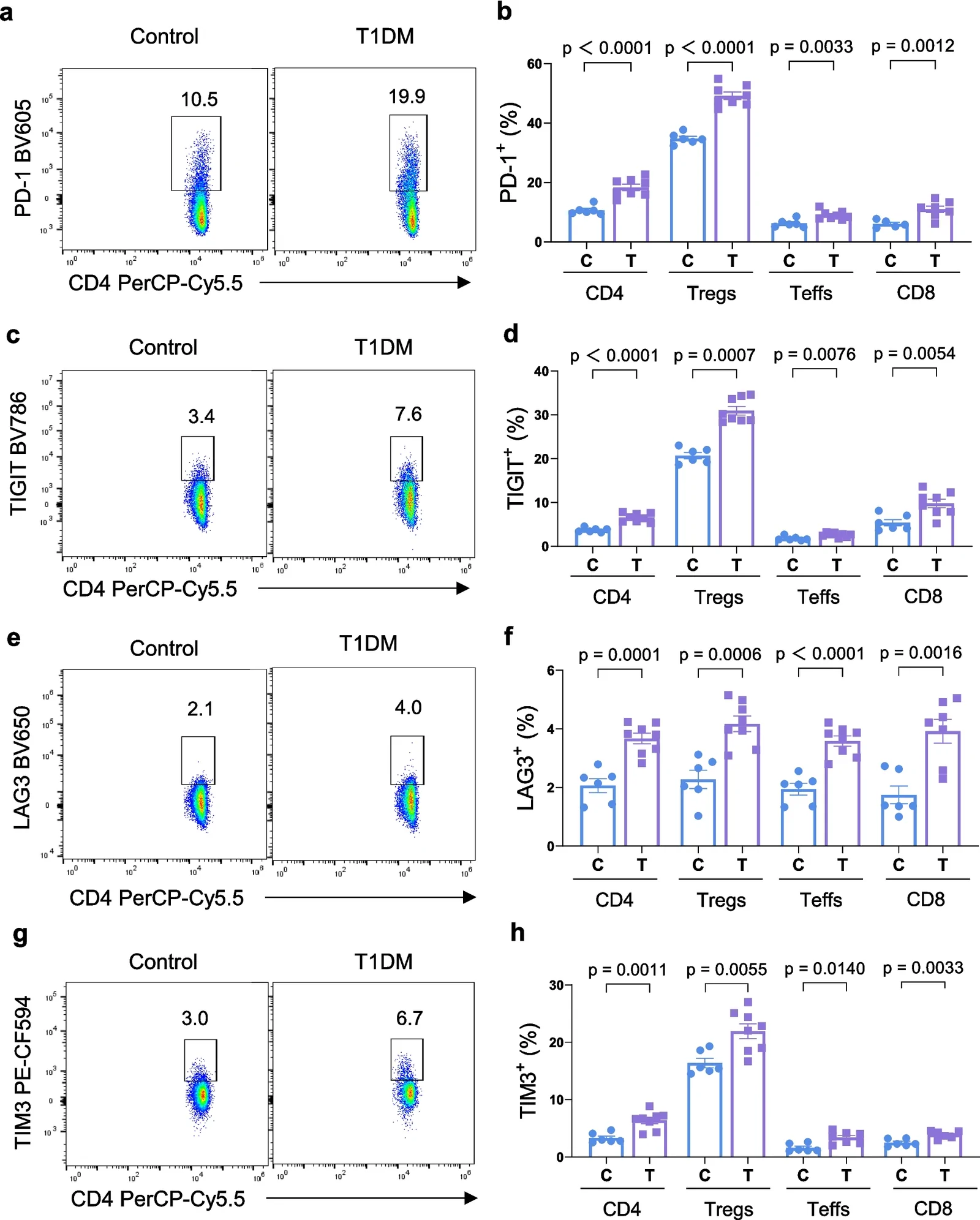
Alterations of PD-1, TIGIT, LAG3, and TIM3 on T cell subsets. **a** & **b** Representative flow cytometry plots and bar graphs of the proportions of PD-1 in CD4^+^ T cells, Tregs, Teffs and CD8^+^ T cells between control and T1DM groups. **c** & **d** Representative flow cytometry plots and bar graphs of the proportions of TIGIT in CD4^+^ T cells, Tregs, Teffs and CD8^+^ T cells between control and T1DM groups. **e** & **f** Representative flow cytometry plots and bar graphs of the proportions of LAG3 in CD4^+^ T cells, Tregs, Teffs and CD8^+^ T cells between control and T1DM groups. **g** & **h** Representative flow cytometry plots and bar graphs of the proportions of TIM3 in CD4^+^ T cells, Tregs, Teffs and CD8^+^ T cells between control and T1DM groups. Error bars represent SEM; *p* values were calculated by two-tailed Student’s t-test

We next examined the expression of the immune checkpoint molecules PD-1, TIGIT, LAG3, and TIM3. The frequencies of each receptor were broadly upregulated across CD4⁺ T cells, Tregs, Teffs, and CD8⁺ T cells in T1DM (Fig. 4). Among the populations examined, Tregs showed marked increases in the proportion of PD-1, TIGIT, and TIM3 in T1DM (Fig. 4). Further analyses revealed that PD-1⁺TIGIT⁺, PD-1⁺LAG3⁺, and PD-1⁺TIM3⁺ Treg subpopulations were also significantly expanded in the context of T1DM (Fig. S7).

These results revealed a relative expansion of CD4^+^CD25^hi^Foxp3^+^ Tregs accompanied by immune-checkpoint expression in STZ-induced diabetic C57BL/6 mice. These findings define a Treg phenotype with high checkpoint expression and sustained immune stimulation in STZ-induced T1DM.

### Suppressive-function-associated markers show subset-specific remodeling in T1DM

**Fig. 5:**
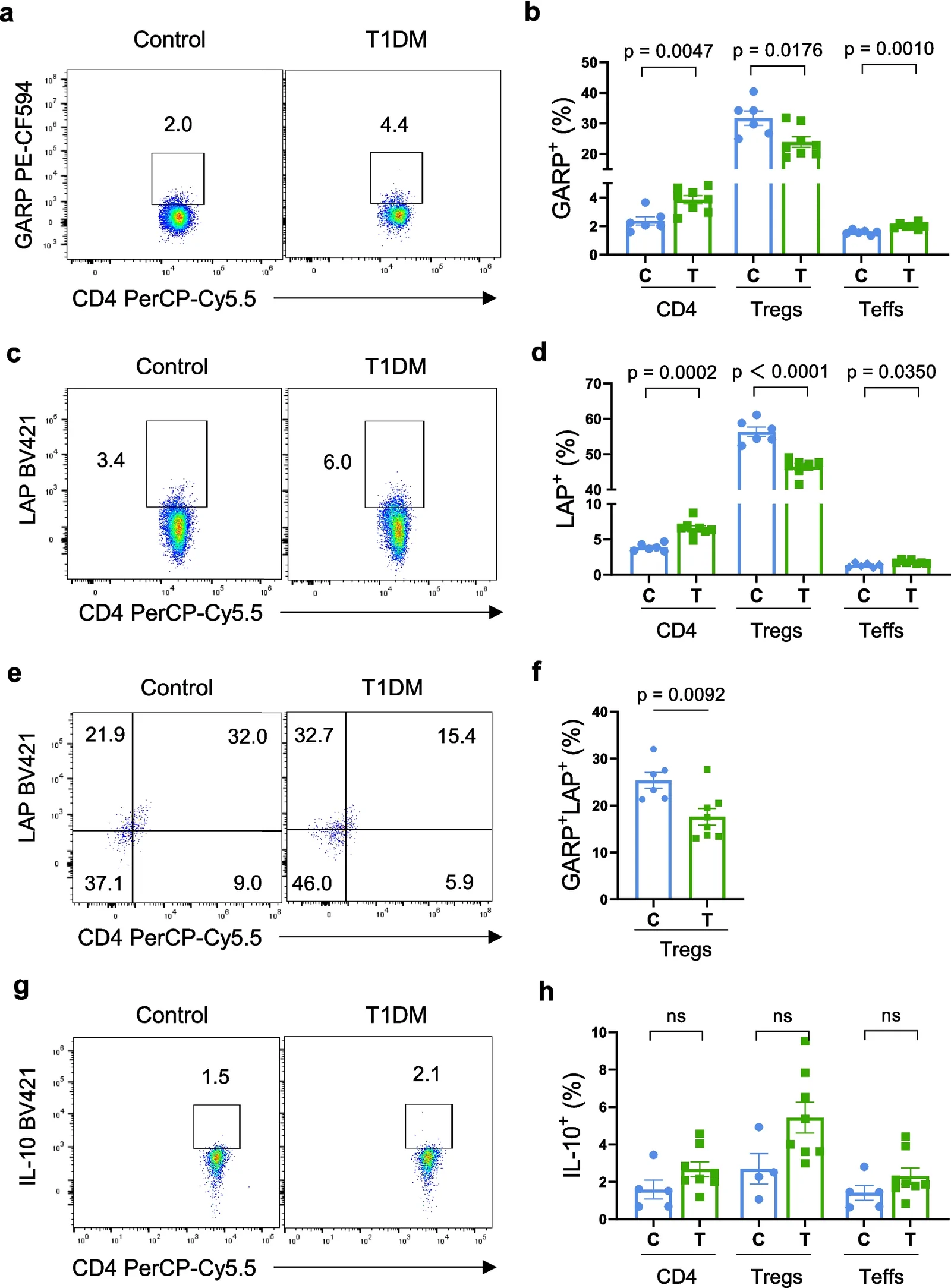
Expression of GARP, LAP, and IL-10 on T cell subsets. **a** & **b** Representative flow cytometry plots and bar graphs of the percentages of GARP in CD4^+^ T cells, Tregs and Teffs. **c** & **d** Representative flow cytometry plots and bar graphs of the percentages of LAP in CD4^+^ T cells, Tregs and Teffs. **e** & **f** Representative flow cytometry plots and bar graphs of the percentages of LAP^+^GARP^+^ in Tregs. **g** & **h** Representative flow cytometry plots and bar graphs of the percentages of IL-10 in CD4^+^ T cells, Tregs and Teffs between control and T1DM groups. Error bars represent SEM; *p* values were calculated by two-tailed Student’s t-test

To characterize suppressive-function-associated phenotypes of Tregs in T1DM, we first examined GARP and LAP, critical components of the TGF-β–dependent regulatory machinery [33]. In T1DM, we found that both GARP and LAP were significantly upregulated in CD4⁺ T cells and Teffs (Fig. 5a-d). Conversely, Tregs in T1DM exhibited a marked reduction in GARP and LAP (Fig. 5a-d), accompanied by a significant decline in the GARP⁺LAP⁺ subset (Fig. 5e & f). These changes were consistent with remodeling of TGF-β-associated regulatory machinery. These findings suggest that although the number of Tregs increased in T1DM, they exhibited reduced expression of inhibitory-function-associated markers. Moreover, we also found that GARP and LAP were preferentially upregulated in CD4⁺Foxp3⁻Helios⁺ and CD4⁺Foxp3⁻Helios⁻ non-Treg subsets, while no significant changes were observed in CD4⁺Foxp3⁺Helios⁺ and CD4⁺Foxp3⁺Helios⁻ Treg subpopulations (Fig. S8).

IL-10 is a key anti-inflammatory cytokine produced by Tregs that contributes to the suppression of effector responses and maintenance of immune tolerance [34]. We observed that the levels of IL-10 in CD4⁺ T cells, Tregs, and Teffs in T1DM showed no significant difference compared to the control group, although an upward trend was observed in T1DM (Fig. 5g & h). Collectively, these findings indicated that Tregs exhibit altered expression of molecules associated with suppressive function, which provides a potential molecular basis for the persistent immune activation and immune tolerance failure in T1DM.

### Skewing toward inflammatory and migratory phenotypes in T-cell subsets in T1DM

**Fig. 6:**
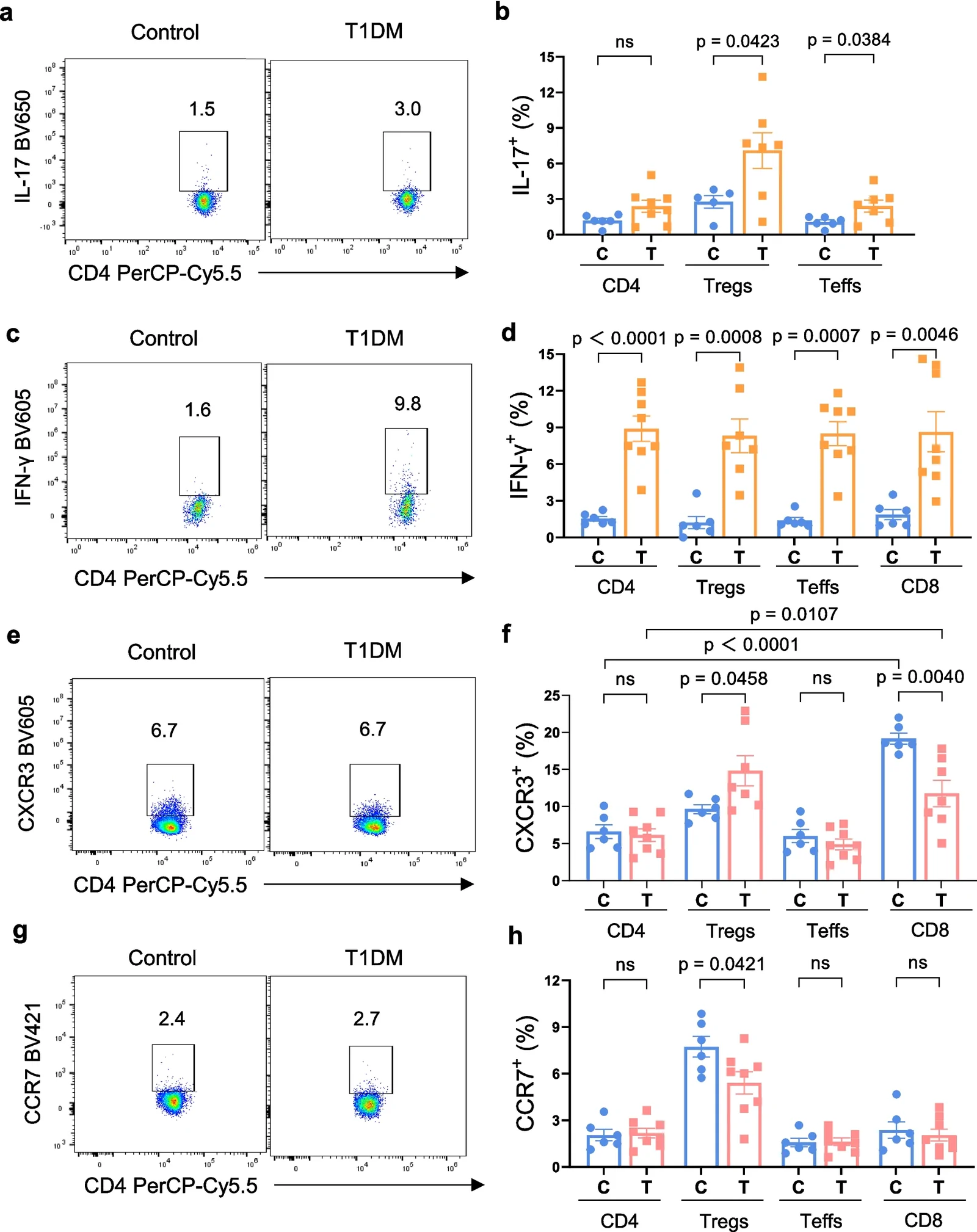
Expression of inflammatory cytokines and chemokine receptors in T cell subsets. **a** & **b** Representative flow cytometry plots and bar graphs of the proportions of IL-17 in CD4^+^ T cells, Tregs, and Teffs. **c** & **d** Representative flow cytometry plots and bar graphs of the proportions of IFN-γ in CD4^+^ T cells, Tregs, Teffs and CD8^+^ T cells. **e** & **f** Representative flow cytometry plots and bar graphs of the proportions of CXCR3 in CD4^+^ T cells, Tregs, Teffs and CD8^+^ T cells. **g** & **h** Representative flow cytometry plots and bar graphs of the proportions of CCR7 in CD4^+^ T cells, Tregs, Teffs and CD8^+^ T cells. Error bars represent SEM; *p* values were calculated by two-tailed Student’s t-test

To investigate inflammatory phenotypes across T cell subsets under diabetic conditions, we measured the frequencies of cytokine-positive cells. Compared with controls, the frequency of IL-17-producing Tregs increased significantly in T1DM, whereas no significant change was detected in total CD4⁺ T cells (Fig. 6a & b). Notably, the proportion of IL-17-producing Tregs was significantly higher than that of IL-17-producing Teffs, suggesting preferential enrichment of IL-17-producing Tregs under diabetic stress. We also observed a significant increase in the frequency of IFN-γ-producing CD4⁺ T cells, Tregs, Teffs, and CD8⁺ T cells (Fig. 6c & d). The acquisition of IFN-γ production by Tregs further indicates a shift from a suppressive phenotype toward an effector-like inflammatory state under diabetic stress.

Next, to explore migration-associated receptor phenotypes of Tregs in T1DM, we examined the expression of chemokine receptors. CXCR3 was significantly upregulated in Tregs, whereas no significant changes were observed in CD4⁺ T cells and Teffs, and it was significantly downregulated in CD8⁺ T cells (Fig. 6e & f) in T1DM. In contrast, the frequency of CCR7⁺ Tregs was significantly decreased, whereas no significant changes were detected among CD4⁺ T cells, Teffs, and CD8⁺ T cells in T1DM (Fig. 6g & h).

### Integrated correlation analysis links transcriptional programs with immune phenotypes and glycemic burden

We first examined the relationships among glycemic burden, Treg/Teff balance, and transcriptional and phenotypic alterations. In Tregs, log_2_(Treg/Teff) positively correlated with blood-glucose AUC and TIGIT expression but negatively correlated with GARP expression, while terminal blood glucose was positively correlated with the frequencies of LAG3^+^ and IFN-γ^+^ Tregs (Fig. S9a). In Teffs, log_2_(Treg/Teff), blood glucose AUC, and LAG3 expression were also positively correlated (Fig. S9b). These results indicated that Treg/Teff balance was associated with increasing glycemic burden and checkpoint- related phenotypes in both Tregs and Teffs in STZ-induced T1DM.

**Fig. 7:**
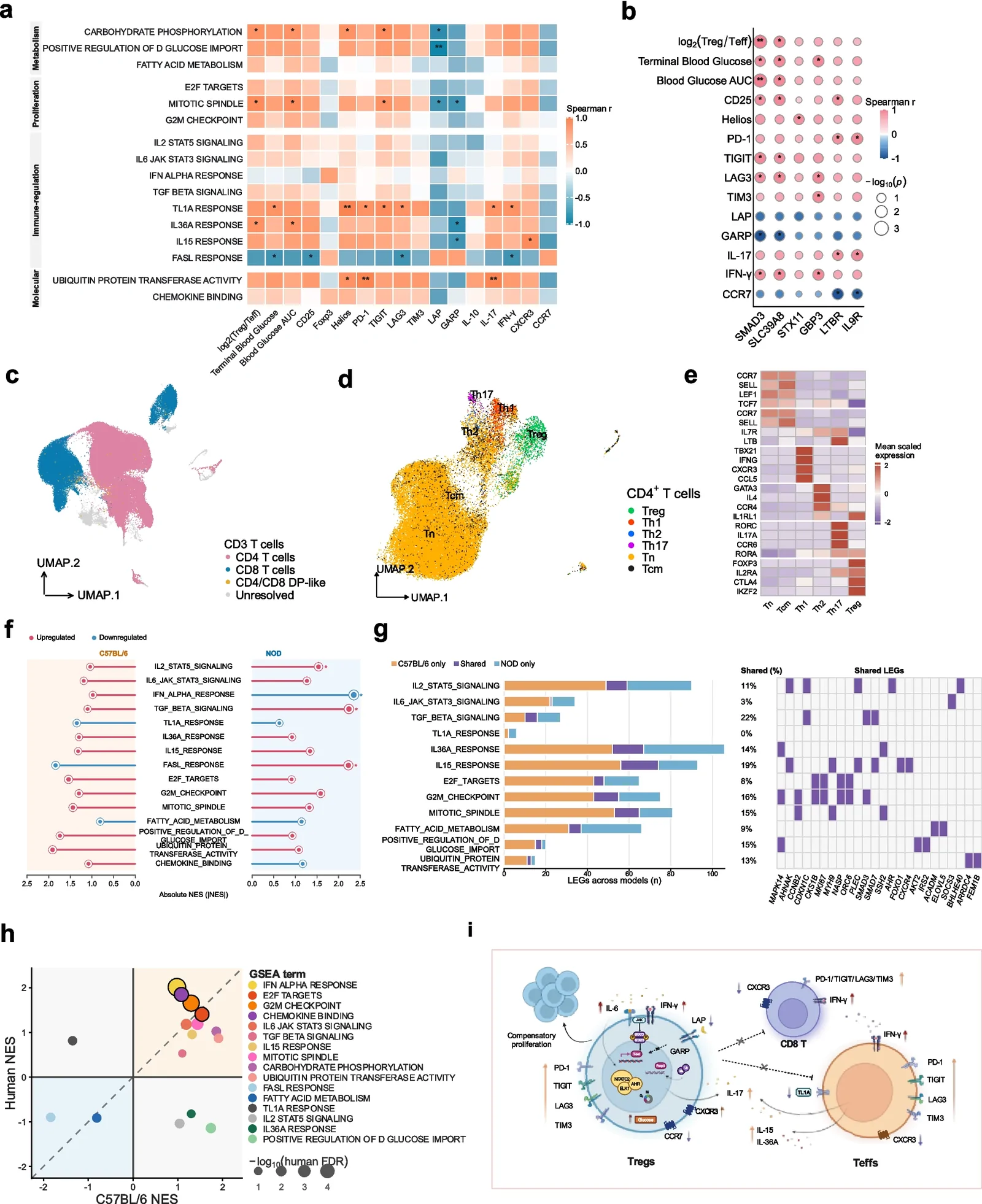
Correlation analysis and cross-model validation in T1DM. **a** Spearman correlation heatmap showing associations between GSVA scores for Treg pathways, and log_2_(Treg/Teff), terminal blood glucose, glucose AUC, and flow-cytometric phenotypes. The color range represents correlation coefficients. **b** Bubble plot showing Spearman correlations between normalized expression of representative Treg identity- and immune-associated LEGs and Treg traits. Bubble color indicates Spearman’s r, and bubble size represents − log_10_(p). **c** UMAP embedding overlaid with transcript-based cell-type annotations of CD3^+^ T cells from GSE200695, classified as CD4^+^ T cells (*n* = 50,042), CD8^+^ T cells (*n* = 25,569), CD4 + CD8^+^ T cells (*n* = 524) or unresolved cells (*n* = 4,405). **d** UMAP visualization of the CD4^+^ T cell subclusters. Cells were classified as Treg (*n* = 1,373), Th1 (*n* = 717), Th2 (*n* = 100), Th17 (*n* = 118), naïve T cells (Tn) (*n* = 31,472) or central-memory T cells (Tcm) (*n* = 3,076) using conservative multi-marker rules. **e** Heatmap showing the scaled expression levels of canonical markers in each cell type. **f** Two-sided lollipop plot comparing GSEA normalized enrichment scores (NES) for Treg-associated pathways in C57BL/6 and NOD Treg datasets. **g** Cross-model LEGs composition across 12 directionally concordant GSEA pathways in C57BL/6 and NOD Treg datasets. **h** Quadrantal diagram illustrating cross-species NES concordance for 16 shared GSEA terms in C57BL/6 and human Treg datasets. **i** Schematic illustration showing the transcriptional and phenotypic alterations of Tregs and Teffs in T1DM. Created with BioRender.com. *p < 0.05, **p < 0.01, ***p < 0.001, ****p < 0.0001

We next assessed associations between enriched pathways, representative LEGs, and glycemic and phenotypic measures. In Tregs, IL-36 A- and TL1A- response pathways were positively associated with log_2_(Treg/Teff), blood glucose AUC, immune checkpoint expression, IL-17 and IFN-γ production (Fig. 7a), while LEGs including *Smad3*, *Slc39a8*, *Gbp3, Ltbr* and *Il9r* showed similar associations and inverse relationships with GARP (Fig. 7b). Metabolic pathways, including carbohydrate phosphorylation and D-glucose import, showed positive associations with Helios, PD-1, TIGIT and LAG3, but negative associations with LAP, with *Hk2*, *Fcsk*, and *Prkci* displaying concordant patterns (Fig. 7a, S9c). Mitotic-spindle enrichment and the E2F-target were positively correlated with blood glucose AUC, and TIGIT expression (Fig. 7a), whereas the G2M-checkpoint *Racgap1* was inversely associated with LAP in Tregs (Fig. S9c). In Teffs, fewer significant associations were observed. IL-36 A-response was positively associated with PD-1, and ubiquitin-protein-transferase activity was positively associated with IL-17 secretion (Fig. S9d). Inflammatory and cytotoxic LEGs, including *Cmpk2*, *Csf2*, *Dntt*, *Adam8*, and *Stx11*, were inversely correlated with log_2_(Treg/Teff) (Fig. S9e).

These analyses linked blood glucose levels and Treg/Teff imbalance to coordinated immune, metabolic, and cell-cycle remodeling. Tregs showed checkpoint and inflammatory, metabolic and proliferative programs, alongside reduced GARP expression, whereas Teffs exhibited fewer, heterogeneous associations involving inflammatory and cytotoxic programs. Overall, these findings reveal coordinated immune–metabolic remodeling of Tregs and Teffs in STZ-induced T1DM.

### Cross-model analyses support conserved Treg transcriptional programs in T1DM

To assess the generalizability of our findings in STZ-induced T1DM, we reanalyzed 2 independent transcriptomic datasets. GSE200695 contains peripheral-blood single-cell RNA-seq (scRNA-seq) data from 4 non-diabetic and 6 diabetic NOD mice, while GSE112341 contains peripheral-blood Tregs from 5 healthy controls and 6 patients with T1DM.

In GSE200695, uniform manifold approximation and projection (UMAP) analysis separated CD3^+^T cells into transcriptome-defined CD4⁺ and CD8⁺ populations (Fig. 7c), and Tregs were identified within CD4⁺ T cells by co-expression of *Cd4, Foxp3*, *Il2ra*, *Ctla4*, *Ikzf2*, *Tnfrsf18*, and *Tigit* (Fig. 7d). The expression patterns of these related genes also support this annotation (Fig. 7e). Of the 16 pathways identified in Tregs in STZ-induced C57BL/6, 12 showed concordant enrichment directions in Tregs in NOD such as IL-2–STAT5 signaling, TGF-β-signaling, and FasL-response (Fig. 7f; S10a). Among the 12 common pathways, the shared LEGs were particularly enriched in IL-15-response, IL-36 A response, G2M-checkpoint, and mitotic-spindle pathways, including *Socs3*, *Smad3*, and *Mki67* (Fig. 7g).

In the human GSE112341 dataset, 12 GSEA pathways also showed consistent directions with Tregs in STZ-induced C57BL/6 mice (Fig. 7h). The concordant pathways comprised 10 positively enriched programs, such as IFN-α-response, IL6-JAK STAT3 signaling, and G2M-checkpoint, together with negatively enriched FasL-response and fatty-acid-metabolism programs (Fig. 7h; S10b). Moreover, 110 shared LEGs were identified between C57BL/6 and human Tregs (Fig. S11a). Importantly, there were 14 common genes with concordant directions shared across C57BL/6, NOD, and human datasets (Fig. S11b, Fig. S11c). These genes collectively represented cytokine and TGF-β regulation, apoptosis, proliferation, mitochondrial and lipid metabolism, stress adaptation, and cytoskeletal organization.

Together, these cross-species analyses support conserved immune, proliferative, and metabolic remodeling of Tregs in T1DM and, integrated with the phenotypic and glycemic associations, suggesting coordinated dysregulation of Tregs and Teffs (Fig. 7i).

## Discussion

In this study, we integrated blood glucose, transcriptome, phenotypic and correlation analyses to characterize the remodeling of Tregs and Teffs in STZ-induced T1DM. Tregs displayed coordinated changes related to checkpoints, inflammation, proliferation, metabolism and migration, while Teffs showed enhanced checkpoint expression and inflammatory activation. Notably, cross-species validation using public Treg datasets supported that multiple transcriptional features are highly conserved in STZ-induced T1DM, spontaneous autoimmunity and human T1DM contexts.

Hyperglycemia may contribute to the alteration of immune phenotypes of Tregs. Correlation analyses revealed the association between blood glucose and transcriptional and phenotypic characteristics. The blood glucose AUC was positively correlated with the log_2_(Treg/Teff), TIGIT expression but negatively correlated with GARP expression. Terminal blood glucose was also positively associated with LAG3 and IFN-γ expression in Tregs. Collectively, these findings indicate that higher blood glucose load was associated with relative Treg expansion and phenotypic alterations, characterized by high checkpoint expression, and a decrease in the GARP level of Tregs in STZ-induced T1DM.

A central finding was the relative expansion of the Tregs, accompanied by broad upregulation of PD-1, TIGIT, LAG3, and TIM3, in STZ-induced T1DM. The increased Treg/Teff ratio and its association with glycemic burden suggest that Treg expansion occurs in the context of diabetes-associated immune activation [35, 36]. PD-1 is associated with metabolic stress and cell survival [37, 38], and TIGIT may represent a compensatory immunoregulatory response to inhibit excessive immune activation [39]. LAG3 may indicate sustained antigen-driven stimulation [40], while TIM3 may mark an activated state with reduced lineage stability and regulatory adaptability [41, 42]. Although immune checkpoint blockade strategies have been established in oncology, strategies aimed at regulating immune checkpoint signaling to restore the immune balance of Tregs in T1DM are still limited. In T1DM mice, a monocyte-derived exosome platform (rExo-IgG) was developed to deliver PD-L1 to inflamed islet cells. By activating the PD-1/PD-L1 pathway, it protects β cells from autoimmune attack and promotes the re-polarization of M2 macrophages, providing a dual therapeutic strategy for the early stage of T1DM [43]. In early clinical stages, a LAG-3 agonist antibody (IMP761) and an oral TIM-3 agonist (LPX-TI641) are under evaluation for their safety and tolerability in other inflammatory diseases, such as rheumatoid arthritis and psoriasis. These findings suggest the feasibility of using immune checkpoints as a target for T1DM, but further evidence is required.

Although Tregs exhibited compensatory expansion, they showed marked downregulation of core suppressive-function-associated molecules, including GARP and LAP. Combined with reduced expression of lineage- and stress-associated genes, including *Zeb2*, *Irak3*, *Dusp22*, and *Hspa1a*, these findings indicate remodeling of Treg regulatory machinery and lineage-associated programs. Preclinical studies have shown that *Zeb2* collaborates with epigenetic regulatory factors to stabilize the Foxp3-dependent transcriptional program, and its absence promotes the transformation of Tregs to an effector-like state under inflammatory stress [44]. Alterations of lineage-associated and suppressive-function-associated programs may compromise Treg stability under inflammatory conditions. These molecules may thus be candidate markers for further investigation.

Tregs additionally acquired inflammatory features in STZ-induced T1DM, including increased IL-17, and IFN-γ production and enhanced IL-6-JAK-STAT3, IL-36, IL-15, and TL1A-response signatures. Although these cytokine networks have previously been considered to be related to the disruption of the Treg suppressive state, the clinical therapeutic effects remain limited. For instance, a phase II trial (NCT02443155) showed that anti-IL-21 monotherapy failed to significantly preserve β-cell function and obtained limited effect after treatment cessation in patients with T1DM [45]. Similarly, the anti-IFN-γ antibody fontolizumab failed to achieve significant clinical improvement in the treatment of Crohn's disease [46]. Collectively, targeting a single cytokine may be insufficient to reverse the complex immune dysregulation, underscoring the need for integrated, multi-pathway interventions.

Furthermore, the migratory phenotypes of Tregs in T1DM have also undergone significant changes. Increased CXCR3 may guide Tregs to the inflammatory site and regulate their localization and inhibitory functions in vivo [47–49]. The downregulation of CCR7 may reduce lymphoid tissue residency [50, 51], while the decreased expression of *Itga1* and *Plxna1* may impair the tissue adhesion ability, thereby further causing Tregs to leave the stable microenvironment [52]. Together with chemokine-related changes in Teffs, these findings suggest potential redistribution of T-cell subsets between lymphoid and inflamed tissues.

The TF and PPI analyses provided additional hypotheses. Upregulation of *Nfatc2*, *Elk1*, and *Ahr* in Tregs was associated with predicted networks involving TCR signaling, MAPK activation, immune regulation, proliferation, and metabolic stress responses. In T1DM, insufficient transcriptional regulation of *Ahr* may weaken the function of Tr1 cells, suggesting an imbalance in immune regulation [53]. Notably, preclinical and early clinical evidence has indicated the therapeutic potential of targeting *Ahr* in systemic lupus erythematosus (SLE) [54] and cancer [55]. In our study, elevated *Ahr* seems to represent a compensatory mechanism of Tregs in T1DM. This highlights the importance of the *Ahr* pathway as a promising strategy for restoring immune tolerance in T1DM. We also observed an increase in *Lyn* expression in Tregs in the T1DM group, which is known to regulate the threshold of TCR and cytokine receptor signals [56]. Currently, an open‑label, phase IIa clinical trial (NCT06474598) is underway to investigate optimal dose and preliminary efficacy of MTX228 (Lyn agonist) in newly diagnosed T1DM patients. Collectively, targeting transcriptional activation alone or combination may provide a promising strategy for preserving Treg function in T1DM patients.

Metabolic remodeling was another prominent feature of splenic Treg dysfunction in T1DM. Carbohydrate-phosphorylation and D-glucose-import programs were enriched, with upregulated LEGs including *Hk2, Pfkfb1, Pfkm*, *Fcsk*, *Prkci, and Rhoq*. In contrast, chronic overactivation of glycolysis may destabilize Foxp3 and impair suppressive function of Tregs under inflammatory conditions. Concurrently, although fatty acid metabolism was significantly downregulated in Teffs and only mildly reduced in Tregs, the concomitant upregulation of *Acox1* in Tregs suggests a compensatory response to metabolic stress [57]. Thus, this enhanced glycolysis and loss of fatty acid oxidation pathway are likely to lead to the functional failure and plasticity loss of Tregs in T1DM.

Limitations of this study should be acknowledged. Although STZ-induced T1DM exhibits key features of pancreatic β-cell injury and inflammatory stress, it cannot fully encompass the genetic and environmental heterogeneity of Tregs in human T1DM. Cross-model analyses partially addressed this limitation. These results support conserved inflammatory, proliferative, and regulatory remodeling in Tregs across STZ-induced, spontaneous and human T1DM. However, differences in species, tissue source, disease stage, and sample size may limit direct comparisons across datasets. Therefore, it will be very important to validate human Tregs from peripheral blood mononuclear cells (PBMCs) or pancreatic tissues in future studies. In addition, our analysis mainly focused on splenic T cells, which provided a systemic perspective on immune dysregulation. However, pancreatic-infiltrating T cells and pancreatic-draining lymph nodes may have distinct transcriptional and functional characteristics. Future studies should continue to explore Treg responses in pancreatic tissue and draining lymph nodes and combine with antigen-specific phenotypic analysis and functional validations.

In conclusion, there was a coordinated remodeling of splenic Treg and Teff transcriptional and phenotypic states in STZ-induced T1DM. The number of Tregs has increased, accompanied by changes in elevated checkpoint, inflammation, proliferation, metabolism and reduced inhibitory characteristics, while Teffs displayed activation related to inflammation and checkpoint expression. Consistent results in NOD mice and human datasets also supported the reproducibility of these features, providing candidate molecular programs and genes for further mechanistic and translational research in T1DM.

## Materials and methods

### Mice

Male C57BL/6 N mice (6–8 weeks) were purchased from the Cam-Su Genomic Resource Center (Jiangsu, China). All mice were housed in a specific pathogen-free (SPF) animal facility under controlled environmental conditions (20–26 ℃; 40–70% humidity; 12-h light/dark cycle) and were given a standard diet and tap water for about 1 week prior to further experiments.

### Type 1 diabetes mellitus induction and assessment

Mice were randomly assigned to either the T1DM group (*n* = 8) or the control group (*n* = 6). Mice in the T1DM group were intraperitoneally (i.p.) injected with STZ (Sigma-Aldrich, St. Louis, MO, USA) at a dose of 50 mg/kg dissolved in cold 0.01 M citrate buffer (Macklin, Shanghai, China) after 4–6 h of fasting for 5 consecutive days [58]. Meanwhile, mice in the control group received an equivalent volume of citrate buffer. Fasting blood glucose was measured from tail vein blood using a glucometer (Sinocare, Hunan, China) on days 1, 4, 5, 6, 9, and 13 after the first STZ injection. The day 13 measurement was performed immediately before tissue collection. Diabetes was defined as a blood-glucose concentration exceeding 13.9 mmol/L on two consecutive days. Mice were euthanized, and tissues were collected on day 13 after the first STZ injection.

### Splenocyte collection

Following euthanasia, spleens were immediately collected in cold phosphate-buffered saline (PBS) (Cytiva, Logan, Utah). Then single-cell suspensions were obtained by gently grinding each spleen through a 40 μm sterile cell strainer. Cells were washed and resuspended in RPMI 1640 medium (Gibco, San Diego, CA, USA) with 10% fetal bovine serum (FBS) (Zeta life, Adams, USA). After cell counting, the splenocytes were frozen in freezing medium (50% RPMI 1640, 40% FBS and 10% DMSO) and stored at − 150 ℃ until further analysis.

### Cell sorting

Single-cell suspensions of splenocytes were resuspended in cold PBS and stained with anti-CD3, anti-CD4, and anti-CD25 (as listed in Supplementary Table 1). Tregs were sorted as CD3^+^CD4^+^CD25^hi^, and Teffs were sorted as CD3^+^CD4^+^CD25^lo/−^ using a FACS Melody (BD Biosciences, New Jersey, USA), with purity exceeding 99.0%. The sorted cells were collected in FACS buffer (cold PBS containing 2% FBS), followed by centrifugation, and cells were lysed with lysis buffer for subsequent bulk RNA-seq.

### Bulk RNA-seq

Tregs and Teffs were sorted from three mice of each group, and cDNAs were synthesized using the Discover-sc® WTA Kit V2 (Vazyme, Nanjing, China) according to the manufacturer’s instructions (v21.1). Sequencing libraries were subsequently constructed using the TruePrep® DNA Library Prep Kit (Vazyme, Nanjing, China) for Illumina and paired-end 150 bp sequencing was performed on an Illumina NovaSeq 6000 system. Raw sequencing reads in FASTQ format were processed using Cutadapt (v1.9.1) to remove technical adapter sequences and low-quality bases. The cleaned reads were aligned to the mouse reference genome using the splice-aware aligner HISAT2 (v2.2.1). Gene-level expression counts were quantified using HTSeq (v0.6.1). Principal component analysis (PCA) was performed using the prcomp R function on the log_2_-transformed normalized counts, after removing batch effects using the removeBatchEffect function in R. Sample-to-sample Pearson correlation coefficients were also calculated using batch-corrected, rlog-transformed count data, and the resulting correlation matrix was visualized as a heatmap using the heatmap package in R.

### Databases for cross-species validation

Public NOD mouse peripheral-blood scRNA-seq database (GSE200695) and human bulk RNA-seq database (GSE112341) were used for cross-model and cross-species validation. GSE112341 includes purified peripheral-blood effector-memory Tregs from 6 adolescents aged 13–16 years with recent-onset T1DM (within one year of diagnosis) and 5 age-matched healthy controls. HbA1c at diagnosis ranged from 9.8% to 15.3% in the T1DM group. These datasets were used to evaluate consistency in the direction of transcriptional alterations and conservation of pathway-level programs, rather than to compare expression levels directly across datasets.

### Differentially expressed gene (DEG) and functional enrichment analysis

DEG analysis was performed on count data using DESeq2 (v1.50.2) after removing batch effects. Genes were considered as significantly differentially expressed if they had an adjusted *p* value (false discovery rate (FDR) < 0.05 and |log_2_ fold change (FC)| > 1. To identify enriched biological pathways, gene set enrichment analysis (GSEA) was conducted on normalized counts using the GSEA software (v4.4.0) [59, 60] and clusterProfiler package (v4.18.4) in R. All gene sets, including mouse-ortholog hallmark, canonical pathways, predicted TF binding sites, gene ontology, and immunologic signature gene sets, were obtained from the Molecular Signatures Database (https://www.gsea-msigdb.org/gsea/msigdb/). To further explore pathway-level alterations, gene set variation analysis (GSVA) [61] was performed on the normalized expression matrix using the GSVA package in R, estimating the relative enrichment of predefined gene sets across individual samples. Data visualization was primarily performed using the packages described above or with ggplot2 (v4.0.1), heatmap (v1.0.13), and ggpubr (v0.6.2) functions in R.

### TF network analysis

The genes encoding TFs in the regulation of Tregs and Teffs were identified using the TRRUST database (www.grnpedia.org/trrust). Data visualization was performed using igraph (v2.2.1) and ggraph (v2.2.2) in R.

### PPI analysis

PPI networks were constructed using the online STRING database for Mus musculus (https://cn.string-db.org/). Functional associations with a combined confidence score ≥ 0.70 were retained. Networks were clustered using the MCL algorithm with an inflation parameter of 3 and visualized in R. Edge widths scaled with STRING confidence scores, whereas solid and dotted edges indicated within- and between-cluster associations, respectively. Four functional groups were annotated for visualization independently of MCL membership.

### Flow cytometry

For surface-marker staining, cells were first incubated with an Fc receptor block (Miltenyi Biotec, Cologne, Germany) for 15 min to prevent non-specific binding. Subsequently, cells were stained for 30 min at 4 ℃ in the dark with different combinations of the following viability dye and mAbs: anti-CD3, anti-CD4, anti-CD8, anti-CD25, anti-PD-1, anti-TIGIT, anti-LAG3, anti-TIM3, anti-LAP (TGF-β1), anti-GARP, anti-CXCR3, anti-CCR7, as listed in Supplementary Table 1. For intracellular staining, cells were fixed and permeabilized with the Foxp3/Transcription Factor Staining Buffer Set (Invitrogen, Carlsbad, CA, USA), followed by staining with anti-Foxp3, anti-Helios, anti-IL-10, anti-IL-17, or anti-IFN-γ, as listed in Supplementary Table 1. For intracellular cytokine staining, cells were stimulated with the anti-CD3/CD28 (Thermo Fisher Scientific, Waltham, MA, USA) for 20 h at 37 °C with 5% CO_2_. Brefeldin A (Thermo Fisher Scientific, Waltham, MA, USA) was added during the final 4 h of stimulation to block cytokine secretion. Rat serum (Senbeijia, Nanjing, China) was added prior to intracellular staining to further reduce nonspecific binding.

Compensation was performed using single-stained compensation beads, and fluorescence-minus-one (FMO) controls were used to establish marker-specific positivity thresholds (Fig. S6). The same thresholds were applied consistently across samples within each staining panel. Cells were acquired on a FACSCelesta flow cytometer (BD Biosciences, New Jersey, USA), and data were analyzed using FlowJo v10.8 (BD Biosciences, New Jersey, USA). For analysis, lymphocytes were identified by FSC and SSC, followed sequentially by singlet, live-cell, and CD3⁺ T-cell gating. CD3⁺ T cells were then classified into CD4⁺ and CD8⁺ subsets; Tregs were defined as CD3⁺CD4⁺CD25^hi^Foxp3⁺ T cells, and Teffs as CD3⁺CD4⁺CD25^lo/−^ T cells. Events below the corresponding FMO-defined threshold were classified as marker-negative, and values near 0% were interpreted as background-level or undetectable signals rather than negative cell frequencies.

## Statistical analysis

Statistical analyses were performed in R (v4.5.2) for RNA-seq data. DEG analysis was conducted with DESeq2 based on the negative binomial distribution and Wald test, with adjusted *p*-values for multiple testing using the Benjamini–Hochberg method to control the FDR. Correlation analyses were performed using Pearson’s correlation or Spearman’s correlation coefficients as appropriate. Flow cytometry data were analyzed using GraphPad Prism v10 (GraphPad software, La Jolla, California, USA) and presented as the mean ± standard error of the mean (SEM). Normality was assessed using the Shapiro–Wilk test. Quantitative differences between two groups were analyzed using an unpaired, two-tailed Student’s t-test. Statistical significance was indicated as follows: * *p* < 0.05, ** *p* < 0.01, *** *p* < 0.001, **** *p* < 0.0001.

## Supplementary Information

Supplementary Material 1.

## Data Availability

The raw and processed RNA-seq data generated in this study have been deposited in the NCBI Gene Expression Omnibus (GEO) database (accession no. GSE350087). Any further data supporting the findings of this study are available from the corresponding author on reasonable request. Additionally, publicly available datasets utilized in this study were obtained from the NCBI GEO database under the accession numbers GSE200695 and GSE112341.

## Abbreviations

AUC: Area under the curve
Ahr: Aryl hydrocarbon receptor
CCR7: C-C motif chemokine receptor 7
CXCR3: CXC chemokine receptor 3
DEG: Differentially expressed gene
FACS: Fluorescence-activated cell sorting
FasL: Fas ligand
FBS: Fetal bovine serum
FDR: False discovery rate
Foxp3: Forkhead box p3
FSC: Forward scatter
Helios/IKZF2: Ikaros family zinc finger 2
GARP: Glycoprotein A repetitions predominant
GEO: Gene Expression Omnibus
GO: Gene ontology
GSEA: Gene set enrichment analysis
GSVA: Gene set variation analysis
IFN-γ: Interferon-γ
IL: Interleukin
LAG3: Lymphocyte-activation gene 3
LAP: Latency-associated peptide
LEGs: Leading-edge genes
Log_2_FC: Log_2_ fold change
MAPK: Mitogen-activated protein kinase
MCL: Markov clustering algorithm
NES: Normalized enrichment score
PBMCs: Peripheral blood mononuclear cells
PBS: Phosphate-buffered saline
PCA: Principal component analysis
PD-1: Programmed cell death protein 1
PPI: Protein‑protein interaction
RNA-seq: RNA sequencing
SLE: Systemic lupus erythematosus
SPF: Specific pathogen-free
SSC: Side scatter
STZ: Streptozotocin
T1DM: Type 1 diabetes mellitus
TCR: T-cell receptor
Teffs: Effector T cells
TF: Transcription factor
TIGIT: T cell immunoreceptor with Ig and ITIM domains
TIM3: T-cell immunoglobulin and mucin domain-containing protein3
Tregs: Regulatory T cells
TL1A: Tumor necrosis factor-like ligand 1A
UMAP: Uniform manifold approximation and projection
