# Supplementary material for: Transcriptional and phenotypic alterations of regulatory and effector T cells in type 1 diabetes mellitus
Source: Mol Biomed. 2026 Oct 24;7:227. doi: 10.1186/s43556-026-00636-9 (PMC13694698; doi:10.1186/s43556-026-00636-9)
Supplement: Supplementary file 1 [file 43556_2026_636_MOESM1_ESM.docx]

**Supplementary Material**

**Title:**

Transcriptional and phenotypic alterations of regulatory and effector T cells in type 1 diabetes mellitus

**Authors:**

Yutong Li^1^, Xinhui Zhao^1^, Mingxin Xie^1^, Eyad Elkord^1,2,*^

**Authors’ Affiliations:**

^1^Centre of Excellence for Oncology and Immunology, Academy of Life and Natural Sciences, Xi'an Jiaotong-Liverpool University, Suzhou, China.

^2^Biomedical Research Center, School of Science, Engineering and Environment, University of Salford, Manchester M5 4WT, UK.

*** Corresponding author:**

Prof. Eyad Elkord

Centre of Excellence for Oncology and Immunology, Academy of Life and Natural Sciences, Xi’an Jiaotong-Liverpool University, No. 111 Ren’ai Road, Suzhou, China, 215123.

Email: [eyad.elkord@xjtlu.edu.cn](mailto:eyad.elkord@xjtlu.edu.cn) or [e.elkord@salford.ac.uk](mailto:e.elkord@salford.ac.uk)

**Fig S1 Alteration of weight and correlation with blood glucose in C57BL6 mice. a** Line graphs showing the longitudinal change in weight for control (n = 6) and T1DM (n = 8) mice. The points and error bars represent the mean ± SEM. **b**. Box plot showing weight AUC of control and T1DM mice. **c** Scatter plot showing the association between blood-glucose AUC and body weight AUC in control and T1DM groups, with each point representing one mouse.

**Fig S2 Heatmap of sample correlation and DEGs in C57BL6 mice.** **a** Sample to sample correlation heatmap depicting the overall transcriptomic similarity among Tregs and Teffs derived from control and T1DM mice. **b** Heatmap displaying expression patterns of significant DEGs across individual samples. The color range reflects the z-score of the normalized gene expression levels from high (red, +3) to low (blue, −3).

**Fig S3 GSEA curves in C57BL/6 mice. a** GSEA curves showing the enrichment of IL2_STAT5_SIGNALING gene set in four comparison groups. b GSEA curves showing the enrichment of IL15_RESPONSE signature in four comparison groups.

**Fig S4 TF and PPI analysis in C67BL6 mice. a** Quadrant plot of TFs among significant DEGs. Each dot represents one TF, positioned according to its log₂ fold change in Teffs from T1DM versus control mice (x-axis) and Tregs versus Teffs from T1DM mice (y-axis). **b** Circle network showing predictive cooperative TFs with three key TFs of Tregs. **c & d** Venn diagrams illustrating overlap genes between downregulated DEGs and LEGs from GSEA in Tregs and Teffs. **e** PPI network of genes downregulated in Tregs and Teffs in T1DM, comprising 103 STRING interactions (combined score ≥0.70); node outlines indicate lineage, edge width indicates interaction confidence, edge style indicates MCL clustering, and ellipses denote visualization-only functional annotations.

**Fig S5 Gating strategy of flow cytometry. a** Lymphocytes were first gated based on forward and side scatter (FSC and SSC), followed by gating of singlet and live cells. CD3⁺ T cells were then identified and further subdivided into CD8⁺ and CD4⁺ T-cell populations. Representative downstream analyses such as IFN-γ, PD-1 and CD25 in CD8⁺ T cells, and CD25, CCR7, Helios, Foxp3 in CD4⁺ T cells. Tregs were identified as CD3^+^CD4^+^CD25^hi^Foxp3^+^ T cells, and Teffs were identified as CD3^+^CD4^+^CD25^lo/-^ T cells. Surface and intracellular markers, including CXCR3, LAP, and TIM-3 in Tregs and IL-17, LAG-3, TIGIT, and GARP in Teffs, are shown.

**Fig S6 Fluorescence-minus-one (FMO) controls for flow cytometry. a** Representative FMO controls for surface markers in flow cytometry, such as CD25, PD-1, TIGIT, LAG-3 and TIM-3, and representative FMO controls for intracellular markers in flow cytometry such as Foxp3, Helios, IFN-γ, IL-10 and IL-17.

**Fig S7 Co-expression of PD-1 with TIGIT, LAG3, and TIM3 on T cell subsets. a** Representative flow cytometry plots and bar graphs of the proportions of PD-1^+^TIGIT^+^, PD-1^+^TIGIT^-^, PD-1^-^TIGIT^+^, PD-1^-^TIGIT^-^ in CD4^+^ T cells and Tregs**. b** Representative flow cytometry plots and bar graphs of the proportions of PD-1^+^LAG3^+^, PD-1^+^LAG3^-^, PD-1^-^LAG3^+^, PD-1^-^LAG3^-^ in CD4^+^ T cells and Tregs. **c** Representative flow cytometry plots and bar graphs of the proportions of PD-1^+^TIM3^+^, PD-1^+^TIM3^-^, PD-1^-^TIM3^+^, PD-1^-^TIM3^-^ in CD4^+^ T cells and Tregs in control and T1DM groups. Error bars represent SEM; p values were calculated by two-tailed Student’s t-test. *p < 0.05, **p < 0.01, ***p < 0.001, ****p < 0.0001.

**Fig S8 Expression of LAP and GARP on Tregs and Teffs.** **a-h** Representative flow cytometry plots and bar graphs of the percentages of LAP and GARP in CD4^+^Foxp3^+^Helios^+^T cells **(a & b),** CD4^+^Foxp3^+^Helios^-^ T cells **(c & d)**, CD4^+^Foxp3^-^Helios^+^ T cells **(e & f)**, and CD4^+^Foxp3^-^Helios^-^ T cells **(g & h)**. Error bars represent SEM; p values were calculated by two-tailed Student’s t-test. *p < 0.05, **p < 0.01.

 **Fig S9 Integrated correlation analysis linking transcriptional alterations, phenotypes and glycemic traits. a & b** Bubble plot showing Spearman’s rank correlations separately in Tregs (a) and Teffs (b). **c** Bubble plot showing Spearman correlations between normalized expression of representative Treg metabolic-associated LEGs and Treg traits. Bubble color indicates Spearman’s r, and bubble size represents −log₁₀ (p). **d** Spearman correlation heatmap showing associations between GSVA scores and Teff traits. **e** Heatmap showing the Spearman’s rank correlation between the expression of LEGs and Teff traits. The color range represents correlation coefficients. *p < 0.05, **p < 0.01, ***p < 0.001.

 **Fig S10 GSEA curves in public datasets. a** GSEA curves showing the alterations of IL2_STAT5_SIGNALING, TGF_BETA_SIGNALING, FASL_RESPONSE, IFN_ALPHA_RESPONSE in the NOD mice database (GSE200695). **b** GSEA curves showing the alterations of IFN_ALPHA_RESPONSE, E2F_TARGETS, G2M_CHECKPOINT, CHEMOKING_BINDING in the human database (GSE112341).

**Fig S11 Cross-model validations of Tregs among the C57BL/6 mice, NOD mice and humans.** **a** Stacked bars show pathway-specific numbers of C57BL/6-only, shared and human-only LEGs, with percentages indicating the proportion shared; the accompanying matrix displays the distribution of shared LEGs across the corresponding pathways. **b** Venn diagram showing the overlap of Treg-associated genes identified in C57BL/6 mice, NOD mice and human datasets, highlighting 14 cross-species shared candidate genes. **c** Forest plot showing log₂FC comparing diabetic with control Tregs for 14 shared candidate genes across three models. Points indicate effect estimates, with red and blue denoting positive and negative effects, respectively. Horizontal bars represent 95% confidence intervals. Shaded bands group genes as immune/regulatory (orange), metabolic/stress (purple) or proliferation/cell-cycle related (green). *p < 0.05, **p < 0.01.

**Supplementary Table 1. List of antibodies used for flow cytometry**

| **Marker** | **Fluorochrome** | **Staining** | **Clone** | **Provider** | **Catalog Number** |
| --- | --- | --- | --- | --- | --- |
| CD3 | BV510 | Surface | 17A2 | BioLegend | 100234 |
| CD4 | PerCP-Cy5.5 | Surface | RM4-5 | eBioscience | 45-0042-82 |
| CD8 | BB515 | Surface | 53-6.7 | eBioscience | 53-0081-82 |
| CD25 | BV421 | Surface | PC61.5 | eBioscience | 404-0251-82 |
| CD25 | BV650 | Surface | PC61 | BioLegend | 102038 |
| PD-1 | BV605 | Surface | J43 | eBioscience | 406-9985-82 |
| TIGIT | BV786 | Surface | TgMab-2 | BD | 744215 |
| LAG3 | BV650 | Surface | C9B7W | BioLegend | 125227 |
| TIM3 | PE-CF594 | Surface | B8.2C12 | BioLegend | 134014 |
| LAP (TGF-β1) | BV421 | Surface | TW7-16B4 | BioLegend | 141408 |
| GARP | PE-CF594 | Surface | F011-5 | BioLegend | 142914 |
| CXCR3 | BV605 | Surface | S18001A | BioLegend | 155915 |
| CCR7 | BV421 | Surface | 4B12 | BioLegend | 120120 |
| Foxp3 | APC | Intracellular | FJK-16s | eBioscience | 17-5773-82 |
| Helios | PE | Intracellular | 22F6 | eBioscience | 12-9883-42 |
| IL-10 | BV421 | Intracellular | JES3-9D7 | BD | 566295 |
| IL-17 | BV650 | Intracellular | eBio17B7 | eBioscience | 416-7177-82 |
| IFN-γ | BV605 | Intracellular | XMG1.2 | BioLegend | 505840 |
